# A forgotten frontier: spinal cord stimulation for iatrogenic and traumatic peripheral neuropathic pain

**DOI:** 10.3389/fpain.2025.1661520

**Published:** 2025-11-13

**Authors:** Linda Kollenburg, Hisse Arnts, Inge Arnts, Robert van Dongen, Heinrich Moser, Frank van Haren, Mark van Ooijen, Robert Jason Yong, Christopher L. Robinson, Erkan Kurt

**Affiliations:** 1 Department of Neurosurgery, Radboud University Medical Center, Radboud University, Nijmegen, Netherlands; 2 Department of Anesthesiology, Pain and Palliative Medicine, Radboud University Medical Center, Radboud University, Nijmegen, Netherlands; 3 Department of Anesthesiology, Perioperative, and Pain Medicine, Harvard Medical School, Brigham and Women’s Hospital, Boston, MA, United States; 4 Division of Pain, Department of Anesthesiology and Critical Care, The Johns Hopkins University School of Medicine, Baltimore, MD, United States

**Keywords:** neuropathic pain, peripheral neuropathy, peripheral nerve injury, chronic pain, spinal cord stimulation

## Abstract

**Introduction:**

Peripheral neuropathy (PN) may cause severe, treatment-resistant pain, especially in traumatic and/or iatrogenic cases. In those with insufficient responses to conventional strategies, spinal cord stimulation (SCS) may be a useful treatment option. However, limited research has been performed on SCS for this indication. This study aims to assess the efficacy, satisfaction and safety of SCS in patients with PN caused by traumatic and/or iatrogenic factors.

**Methods:**

Patients with traumatic and/or iatrogenic PN, implanted with SCS between 2005 and 2021 at Radboudumc are included in this study. Perioperative data on efficacy, satisfaction, and safety were retrospectively collected from the electronic patient records (EPIC) and analyzed using descriptive statistics. The efficacy is assessed with the numeric rating score (NRS). Responders are defined as those having ≥50% reduction in NRS.

**Results:**

Fifteen patients (*M* = 8, 48 ± 12 years) are included. At last follow-up (2–18 years), 63% (10/15) of patients are defined responders with an average decrease in NRS of 63% (8.1 ± 0.8 to 3.0 ± 2.0) (*p* < 0.01). All patients are satisfied with their implant. A complication was present in one patient, reporting a superficial infection (6%, 1/15) following implantation.

**Discussion:**

Unlike peripheral nerve stimulation (PNS) and dorsal root ganglion (DRG) stimulation, which are more frequently considered for patients with PN caused by traumatic and/or iatrogenic factors, SCS enables central nervous system stimulation via the spinal cord, thus targeting pain regions associated with multiple lower limb nerve roots. As PN, caused by trauma and/or iatrogenic factors may affect multiple nerves simultaneously, it is suggested that SCS offers improved clinical benefit for these patients.

**Conclusion:**

The current study demonstrates that SCS is a promising treatment modality for patients with traumatic and/or iatrogenic PN. Prospective trials comparing SCS to treatments like PNS and DRG stimulation are essential to substantiate its efficacy, expand its indications, and inform future clinical guidelines for patients with intractable traumatic or iatrogenic peripheral neuropathy.

## Graphical Abstract

**Figure:**
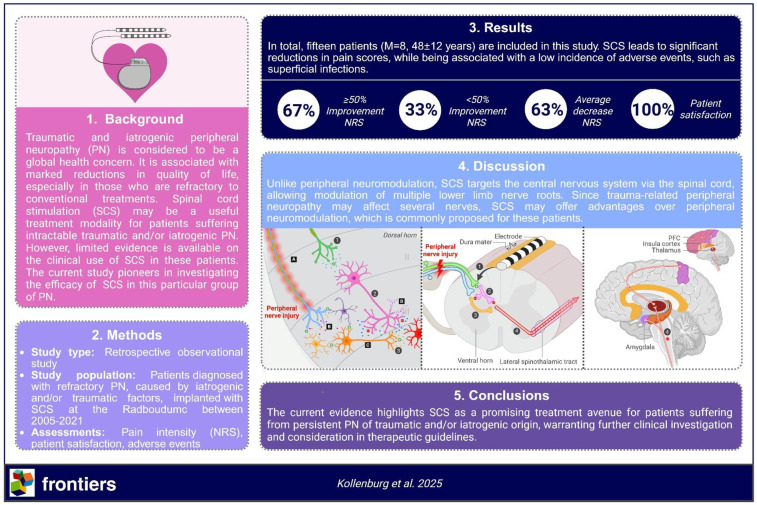

## 1 Introduction

**Figure 1:**
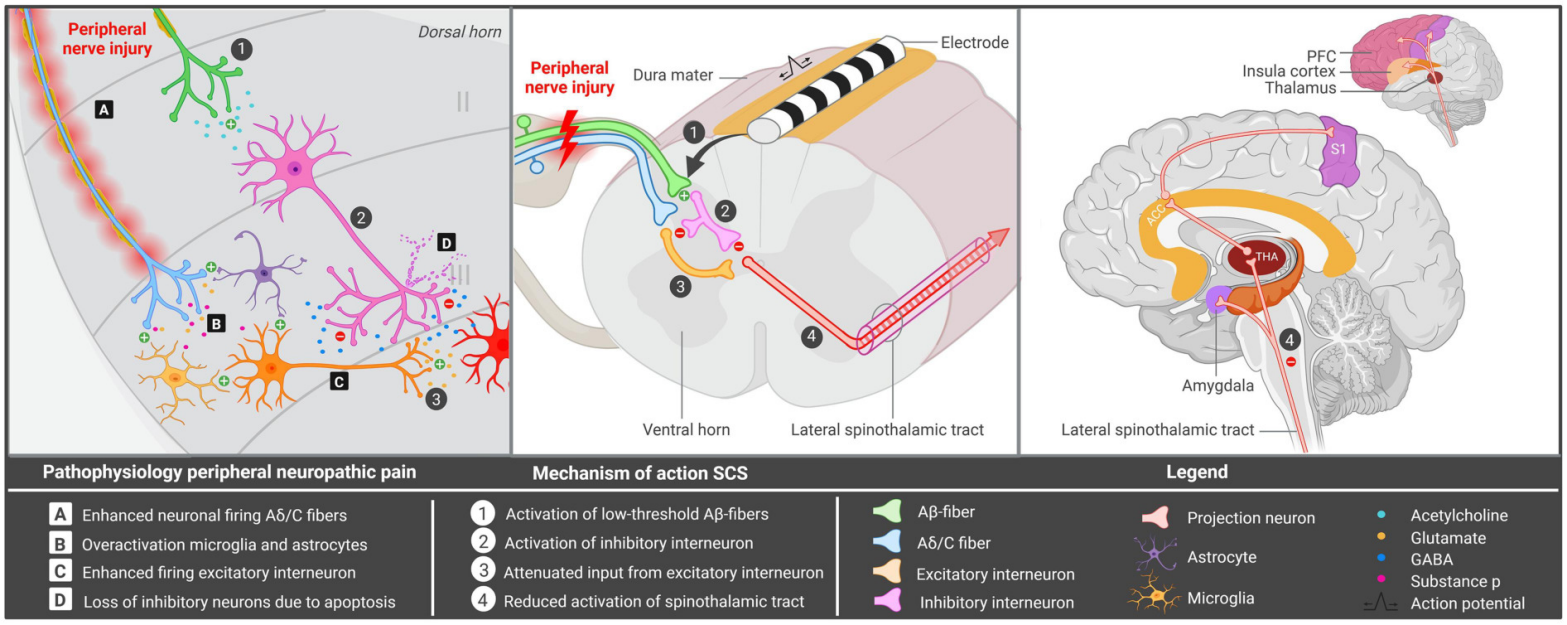
Hypothesized mechanism of spinal cord stimulation for peripheral iatrogenic/traumatic neuropathy. Overview of the mechanism underlying peripheral nerve injury and spinal cord stimulation. ACC, anterior cingulate cortex; GABA, gamma-aminobutyric acid; PFC, prefrontal cortex; SCS, spinal cord stimulation; S1, primary somatosensory cortex; THA, thalamus. Created in BioRender. Kollenburg, L. (2026). https://BioRender.com/mwlweeu.

Peripheral neuropathy (PN) is a significant global health challenge, impacting 7%–10% of the general population (1). It is associated with marked reductions in physical, social and emotional functioning (2) and manifests as burning, tingling and/or stabbing sensations, with pain being present in almost two thirds of patients (3). Common causes of painful PN include diabetes mellitus, complex regional pain syndrome (CRPS) type II, post-amputation or phantom limb pain, and direct trauma or iatrogenic factors (3). Traumatic and/or iatrogenic PN is often difficult to treat, while various pharmacological options, local minimally invasive injections and more invasive alternatives involving peripheral neuromodulation techniques [e.g., peripheral nerve stimulation (PNS) and dorsal root ganglion (DRG) stimulation] have been developed to treat these patients (4, 5). However, a subset of these patients remains refractory to conventional strategies and often suffer psychiatric symptoms like depression, consequently leading to a marked decrease in quality of life (6, 7). The complex pathophysiology of traumatic and/or iatrogenic PN, which may simultaneously affect multiple nerves, likely contributes to treatment resistance. Hence, alternative neurosurgical strategies, such as spinal cord stimulation (SCS) should be considered for this particular group. SCS involves implantation of a lead and an implantable pulse generator (IPG), allowing for electrical stimulation of various structures in the spinal cord. Improvement in pain intensity is thought to be achieved by enhanced activation of low threshold Aβ-fibers, consequently leading to decreased nociception by inhibition of the lateral spinothalamic tract (Figure 1) (8).

Over the years, SCS has shown to be a valuable treatment option for various chronic pain disorders, such as diabetic neuropathy and persistent spinal pain syndrome (PSPS), with an overall response rate of ≥50% (9). Despite promising outcomes, SCS is often not considered a treatment option for patients suffering intractable painful PN caused by iatrogenic and/or traumatic factors. Since 1996, no literature has been published on this subject (10). As a result, there is limited awareness and only a weak recommendation for the use of SCS in painful PN (11). For this purpose, more research is needed to further explore its therapeutic potential. The current study aims to evaluate the efficacy of SCS in patients with refractory painful PN caused by iatrogenic and/or traumatic factors. Outcomes provide valuable insights for interventional pain physicians, serving as a crucial resource for achieving optimal management of patients with intractable traumatic and/or iatrogenic PN.

## 2 Materials and methods

### 2.1 Study population

Patients diagnosed with refractory PN, caused by iatrogenic and/or traumatic factors, implanted with SCS at the Radboudumc between 2005 and 2021, were selected. PN is defined as refractory if insufficient pain relief was obtained following the European federation of neurological societies (EFNS) Step-by-Step treatment [including physiotherapy, transcutaneous electrical nerve stimulation (TENS), optimal medication and different forms of nerve blocks] (12, 13). Patients with <2 years follow-up, and those suffering CRPS type II or PN with other causes than iatrogenic and/or traumatic factors, such as chemotherapy, radiotherapy, pharmacological treatments and thermal injuries, were excluded from this study. In all patients, trial stimulation was performed prior to implantation of the SCS system. Subjects received a similar surgical approach to SCS with an 8-pole electrode (Vectris Octad, Medtronic Inc., Minneapolis, MN, USA or Octrode, Abbott) being implanted percutaneously with the tip placed between T8 and T12. At the time of surgery, there was no reimbursement in the Netherlands for SCS in PN. Hence, prior to surgery, reimbursement was requested for all patients to individual health insurance companies on a case-by-case basis.

### 2.2 Study design and outcomes

The primary aim of this retrospective observational study is to investigate the effect of SCS on pain intensity in patients with intractable PN caused by iatrogenic and/or traumatic factors. The secondary aim of this analysis is to evaluate the overall satisfaction rate and safety of SCS in this group. As part of the standard procedure for SCS at the Radboudumc, patients completed a questionnaire three times daily over four consecutive preoperative days, with pain intensity being evaluated with Numeric Rating scale (NRS). These preoperative data have been collected retrospectively from the electronic patient database of Radboud university medical center (EPIC). At the last follow-up in April 2024, patients were contacted and asked about satisfaction and efficacy of SCS. With regard to satisfaction, patients were asked if they would undergo the surgery again, if answered with yes, patients were classified as satisfied. To evaluate efficacy, postoperative data on pain intensity have been collected at the last-follow up and compared to preoperative outcomes. Responders are defined as those having ≥50% improvement in NRS.

### 2.3 Ethical considerations

This study was performed according to the Dutch law and Ethical Principles for Medical Research Involving Human Subjects, outlined in the World Medical Association's Declaration of Helsinki revised in 2013. The Medical Review Ethics Committee region Arnhem-Nijmegen concluded that this study was not subject to the Medical Research Involving Human Subjects Act (CMO Oost-Nederland; file number: 2024-17523). All patients gave consent for using their data for the current manuscript.

### 2.4 Statistical analysis

Preoperative and postoperative data on the efficacy, satisfaction and safety of SCS in patients with traumatic and/or iatrogenic PN, were analyzed using descriptive statistics, in which all values and differences are compared and described manually. Statistical analyses for the determination of significance in pain scores were performed with SPSS Statistics for Windows (Version 31.0, IBM Corp, Armonk, NY). The Shapiro–Wilk test was used to check if these data were normally distributed. If data were normally distributed, a dependent samples T test was performed to assess statistical significance in pain scores before vs. after treatment with SCS. *P*-values <0.05 are statistically significant.

## 3 Results

Fifteen patients (male = 8, 48 ± 12 years) were included in the current analysis with an average follow-up of 10 (2–18) years (Table 1). Out of all patients, five suffered nerve injury after a trauma and 10 after a surgical intervention of the lower limb for various indications. No pronounced neurovegetative symptoms were present in the study population. The average decrease in NRS is 63% (8.1 ± 0.8 to 3.0 ± 2.0) (*p* < 0.01). In total, 67% (10/15) of patients are defined as responders (Table 1). Two patients requested for removal of the SCS system as their original pain had permanently decreased. Further, all patients (100%, 15/15) are satisfied with their implant. The only biological complication was a superficial infection in one patient, which was treated with antibiotics (6%, 1/15). In total, reoperations are performed in 47% (7/15) of patients. Four patients underwent IPG replacement due to an empty battery (13%, 2/15) or pocket pain (13%, 2/15); in one of the patients with battery depletion, a simultaneous electrode revision was performed. One patient received a revision of the entire SCS system due to defect contacts (7%, 1/15), and in two patients the system was explanted due to permanent pain reduction (12%, 2/15) (Table 1).

**Table 1:** Outcomes of spinal cord stimulation in patients with peripheral neuropathic pain due to trauma and/or iatrogenic factors.

Patient	Age (y)	Gender	Diagnosis	Tip of lead	Follow up	Satisfied	NRS preop	NRS postop	Reduction in NRS (%)	Remarks
1	70	M	Iatrogenic injury of Ischiadic nerve after previous pain treatments	T10	2 y (27 m)	Yes	8	4	50	N/A
2	42	F	Iatrogenic injury of ischiadic nerve after resection of sarcoma in buttocks	T9–T10	7 y (87 m)	Yes	9	6	33	N/A
3	46	M	Traumatic injury of peroneal nerve after bone fracture	T12	14 y (175 m)	Yes	9	5	44	N/A
4	36	M	Iatrogenic injury of genitofemoralic nerve after surgery in inguinal region	T8	18 y (226 m)	Yes	8	4	50	Revision Quad to Octad electrode, revision IPG due to empty battery
5	45	F	Iatrogenic injury of peroneal nerve after surgery on fibula	T11	6 y (72 m)	Yes	9	2	77	N/A
6	47	M	Iatrogenic injury of saphenous nerve after surgery for varices	T9	12 y (149 m)	Yes	8	2	75	Revision IPG due to empty battery
7	27	F	Traumatic injury of ischiadic nerve after bone fracture	T8–T9	17 y (214 m)	Yes	9	6	33	Revision entire SCS system due to defect contacts
8	60	M	Traumatic injury of peroneal nerve after bone fracture	T8	2 y (27 m)	Yes	8	2	75	N/A
9	47	M	Iatrogenic injury of ilionguinal nerve after surgery in inguinal region	T10–T11	14 y (173 m)	Yes	8	0	100	N/A
10	69	M	Iatrogenic injury of ischiadic nerve after surgery of hip	T9–T10	10 y (131 m)	Yes	8	5	43	N/A
11	50	F	Iatrogenic injury of nervus suralis en peroneus after surgery for neuralgia	T10	7 y (87 m)	Yes	7	4	43	Revision IPG due to pocket pain
12	54	M	Iatrogenic injury of peroneal nerve after surgery on fibula	T9	6 y (75 m)	Yes	8	3	63	N/A
13	56	F	Iatrogenic injury of nervus ilioinguinalis after surgery in inguinal region	T8	6 y (72 m)	Yes	6	0	100	Revision IPG due to pocket pain
14	27	F	Traumatic injury of ischadic and peroneal nerve after femur fracture	T10	13 y (156 m)	Yes	9	3	67	SCS system removed after 11 years due to permanent pain reduction
15	54	F	Traumatic injury of ischadic and femoral nerve after pelvis fracture	T8	18 y (219 m)	Yes	8	0	100	SCS system removed after 12 years due to permanent pain reduction, infection

F, female; h, hour; m, months; M, male; N/A; not applicable; T, thoracic vertebra; y, year.

## 4 Discussion

Traumatic and iatrogenic PN has major socioeconomic consequences, as it may cause enhanced health care costs and mental disorders like depression (14). The current study investigates the efficacy, satisfaction and safety of SCS in this group of patients.

### 4.1 Efficacy of SCS for PN

Outcomes of this study indicate that SCS is a potential treatment option for traumatic and/or iatrogenic PN, with 67% of patients being responders. Despite limited research being published on this specific group of PN, the response rate obtained within the current study corresponds with outcomes reported for SCS in other causes of PN. Data suggests improvement in pain intensity in 58%–65% of patients with CRPS (15, 16). Further, response rates of 58%–100% are mentioned for patients with diabetic and idiopathic neuropathy (9, 10, 17). In addition, reports show that SCS is effective in 48%–88% of patients with PN related to persistent spinal pain syndrome related (18, 19). In patients suffering stump pain after amputation and/or phantom limp pain in the lower extremities, authors report response rates of 83% (20) and 100% (21). Promising outcomes of SCS in diabetic neuropathy and/or leg/back pain further support consideration of this strategy in the treatment spectrum of PN caused by trauma and/or iatrogenic factors (19). In the current analysis, 100% of patients are satisfied, which can likely be attributed to all patients experiencing sufficient reduction in pain intensity. The satisfaction rate is higher than previous outcomes on SCS for chronic pain, reporting an overall rate of 82.2% (22).

Discrepancies in outcomes of SCS for various forms of PN may be caused by variability in surgical technique, responder definition, follow-up, stimulation settings and underlying etiologies of PN (23). It is important to note that some outcomes on SCS for painful PN were published further back in time (10), hence why outcomes may differ from more recent articles in which more advanced approaches to SCS have been used. With regard to surgical technique, it can be expected that electrode placement most proximal to the root of the affected nerve(s) will correlate to better outcomes (24). Concerning the follow-up, studies have shown that around 13%–26% of patients undergoing SCS experience loss of efficacy over time due to the presence of habituation, which may be caused by neuronal plasticity (25–28). This phenomenon likely forms an explanation for the loss of responders found in patients with PN receiving SCS on the long-term and may suggest that studies with a longer follow-up period may have decreased response rates (10).

Another important aspect contributing to discrepancies in study outcomes includes variability in etiologies causing PN, as these may interfere differently with SCS. To illustrate, SCS most likely acts upon the myelinated fibers of the gate control system, however, in certain pathologies, peripheral pain is also suspected to be caused by disturbances in other pathways including non-myelinated fibers, which may lead to diminished efficacy of SCS (10). It is noteworthy that, in cases of diabetic PN, it can be considered a continuous and systemic disease which progresses over time, whereas this is not the case for PN caused by nerve injury and/or iatrogenic factors, thus likely affecting outcomes of SCS (29). The importance of variability in etiologies on clinical outcomes of SCS is confirmed by findings of Kumar et al. suggesting lower efficacy of SCS in patients with PN caused by postherpetic neuralgia and intercostal neuralgia when compared to diabetic and idiopathic neuropathy (10). However, as the involvement of chronic pain pathways in various forms of peripheral neuropathic pain is similar, it makes it somewhat questionable to what extent the etiology affects outcomes of SCS (30).

In the current study, it is noteworthy that in two patients, the SCS system was explanted due to permanent reduction in pain intensity, even when stimulation was turned off for a long period of time (13%, 2/15). Though rarely described, the presence of this phenomenon has also been reported for patients with CRPS undergoing SCS (31). Further, a retrospective chart review study similarly reports explant of SCS due to permanent pain reduction in 3/962 patients with various indications of chronic pain (32). Though the mechanism underlying this observation remains unknown, it may be caused by permanent reversal of central sensitization (33), increase in current perception threshold (34), nerve regeneration and/or cortical plasticity (35). Meier et al. investigates the carry-over effect in SCS and attributes the occurrence to “peripherally induced reconditioning of the central nervous system”, suggesting that selective activation of Aa/β fibers could temporarily reverse neuronal hyperexcitability and changes in descending supraspinal circuits induced by chronic pain (36). Though the carry-over effect has only been described to last for several hours, a similar mechanism might be present for permanent disappearance of pain after explant of the SCS system (36). It remains unclear as to why this phenomenon solely occurs in certain patients, however, Lee and colleagues suggest that age may play an important role as young patients are thought to have greater flexibility returning from disturbances in the sympathetic nervous system (31). Further, factors including pain intensity, etiology, stimulation paradigms might also be involved as these are thought to affect the duration of the carry-over effect in SCS (36). Interestingly, despite similar effects on pain reduction, only limited number of patients requested removal of the SCS system. This might be attributed to pain reduction not being permanent, anxiety for regaining painful sensations after the explant or patients not being bothered by their implant. Due to limited data availability, the mechanism of SCS and (permanent) carry-over should be further investigated as it could provide valuable insights for treatment optimization of SCS for PN. Other factors contributing to this phenomenon may be permanent alterations in personal stressors, leading to improvements in pain intensity over time.

A single technical complication, IPG site pain (2/15), occurred in this study and has also been mentioned by others covering SCS for peripheral neuropathic pain (37), alongside other adverse events such as lead migration and lead breakage (9, 20, 38), which did not occur in the current study. Furthermore, only a single biological complication occurred, while others report additional adverse events (AEs) including cardiac arrest, femur fracture, allergic dermatitis, hematoma, and wound dehiscence/impaired healing in patients with peripheral neuropathic pain undergoing SCS (21, 38, 39). Interestingly, the AEs, except for infection (3%–8%), are considered rare for patients with chronic pain undergoing SCS (40). The discrepancy in AE rates between the overall chronic pain- and peripheral neuropathic pain group is likely due to patients with PN often suffering additional comorbidities like diabetes, hence making them more prone to develop infections and other wound related complications (41). Due to an absence of additional comorbidities in the current study population, lower incidences of biological complications were also expected.

### 4.2 Mechanism of SCS in PN

In PN, SCS is hypothesized to alter neurochemical signaling in the dorsal horn, inhibiting central neuronal hyperexcitability in the nociceptive system (8). Peripheral nerve injury enhances neuronal firing via the Aβ and c fibers, leading to excessive release of glutamate and substance P in the dorsal horn (8) (Figure 1). It may also increase apoptosis of inhibitory interneurons, as well as cause hyperexcitability of microglia and astrocytes (42–44). As a result, the secondary order neuron depolarizes, leading to enhanced activation of the spinothalamic tract and increased transmission of pain signals to various cortical and subcortical areas (Figure 1). SCS potentially reduces nociception by inhibiting activity in the spinothalamic tract, through stimulation of Aβ fibers and activation of inhibitory interneurons (33, 34, 45). Following stimulation of these interneurons, opioids are released, leading to the hyperpolarization of Aδ and c fibers as well as secondary order neurons. As a result, pain transmission via the spinothalamic tract is reduced (33). SCS may also enhance GABA release, leading to the suppression of wide dynamic range (WDR) neurons, which are shown to be hyperexcitable after nerve injury (46, 47). Through connections with the Aδ and C fibers, inhibition of WDR neurons likely acts on similar ascending pain pathways (47). Evidence also suggests that SCS terminates wind-up in C fibers, which is a phenomenon describing increased pain intensity over time following repeated nociceptive stimulation (47). SCS has also been shown to counteract the activation of microglia cells, which may play an important role in peripheral neuropathic pain (Figure 1) (48).

### 4.3 Efficacy and mechanisms of peripheral neuromodulation in PN

Unlike SCS, peripheral neuromodulation techniques are more frequently considered as a treatment option for PN. Previous literature has explored the efficacy of conventional neuromodulation techniques, such as PNS, as well as more innovative approaches, like DRG stimulation (49, 50). Previous findings suggest that PNS of the tibial nerve causes 65%, 79% and 83% pain relief after 1, 3 and 6 months follow-up respectively in six patients with PN and CRPS (51). Other studies report a 57.4% reduction in pain intensity at 12-month follow-up following PNS of the brachial plexus in a cohort of ten patients with CRPS (52). Furthermore, authors state that 71% of patients with various forms of chronic pain (including CRPS) experience ≥50% improvement in pain after treatment with 60-day PNS (53). The therapeutic effects of PNS are thought to be established via peripheral and centrally acting mechanisms. On the central level, PNS may reduce hyperalgesia and central sensitization by inhibiting the activity of dorsal horn interneurons and wide dynamic range neurons, and through modulation of GABAergic and serotonergic pathways (50). Peripherally, PNS is theorized to suppress pain signal transmission by modulating large diameter Aβ afferent nerve fibers, without small fiber activation (50). With regard to DRG stimulation and PN, Ege and colleagues investigated the efficacy of this approach in nine patients with chemotherapy-induced PN. They report a mean reduction of 2.3 in average and 2.6 in worst pain using the visual analogue score (54). Notably, pain scores further ceased after 3 months, with the lowest pain scores (average of 1.9) being reported after 6–12 months (54). Others mention an average decrease of 64.2% (*N* = 4) (at 12 months follow-up) (55) and 80% (*N* = 8) (at 6 weeks follow-up) (56) for patients with painful diabetic neuropathy. Data also shows improvements of 67% (*N* = 8) (at 3 days follow-up) (57), 100% (*N* = 1) (at 3 years follow-up) (58), 40% (*N* = 1) (at 20 months follow-up) (59), 71% (*N* = 7)(at 1 week follow-up) (60) and 49% (*N* = 33) (at 12 months follow-up) (61) in those suffering idiopathic PN and polyneuropathy. The precise mechanism behind DRG stimulation in PN remains unclear. However, several theories imply that it may suppress the transmission of pain signals by acting at the T-junction of nociceptive neurons, stimulating postsynaptic activation of pain-gating circuits in the dorsal horn and DRG, and modulating the intrinsic excitability of DRG neurons (62).

### 4.4 SCS vs. peripheral neuromodulation for PN

Literature suggests that only small portion of patients with PN receive SCS, owing to the lack of awareness of SCS guidelines, absence of reimbursement, and low referral rates for this indication (63). The lack of clear indications contributes to limited clinical experience. An unstandardized surgical approach and the inclusion of heterogenous groups likely also contribute to physicians' reluctance to implement SCS for painful PN caused by trauma and/or iatrogenic factors. As a result, clinicians select pharmacotherapy or peripheral neuromodulation techniques (e.g., PNS and DRG stimulation) instead of SCS for patients suffering traumatic and/or iatrogenic PN (62). To date, very limited research has been published on the comparison between SCS and peripheral neuromodulation techniques, especially for PN. It should, however, be emphasized that each neuromodulation technique acts on a different target site and may thus be preferred in specific clinical scenarios. PNS and DRG stimulation may especially be effective when peripheral neuropathic pain originates from a single affected nerve, due to their ability to target areas closely adjacent to the affected nerves. Consequently, it is theorized that in mononeuropathy, lower amplitudes are needed to achieve the desired effect, compared to SCS, where greater current loss may occur across the cerebrospinal fluid (CSF) layer and dura mater (50). In addition, proximity allows for precise modulation, potentially leading to greater reductions in pathological neuronal firing near the origin of pain. Supporting this, studies have demonstrated enhanced GABA release within the dorsal horn of the spinal cord following DRG stimulation, compared to SCS (64, 65). The advantage of precise, localized targeting of the affected nerve in these treatments also presents a limitation, especially in patients with traumatic and iatrogenic PN involving multiple branches originating from different nerve roots (e.g., the sciatic and concomitant peroneal nerves arising from L4–S2–S3), as was the case in the current study sample. In this particular group of PN patients, DRG stimulation and/or PNS would require the placement of multiple electrodes at different spinal levels and/or peripheral nerve branches respectively, making SCS a more practical and comprehensive option. Further, SCS is often also preferred in patients who received multiple surgeries in the affected area as scarring and/or anatomical abnormalities may be present which complicate PNS but not SCS. Moreover, SCS may be particularly advantageous over PNS in cases involving nerves located deep within the lower abdomen and groin region (e.g., ilioinguinal nerve and genitofemoral nerve), where accurate targeting with PNS can be technically challenging. Finally, another advantage of SCS over PNS and DRG, is the ability to automatically adjust stimulation parameters with closed-loop systems during postural changes, allowing consistent efficacy throughout bodily movement (66).

### 4.5 Strengths and limitations

Though the sample size of the current study is relatively small, due to lack of reimbursement, it does remain the largest sample size of studies investigating SCS in patients with PN caused by trauma and/or iatrogenic factors. Additionally, the length of follow up in the current sample is also considered a major strength. Whereas a subset of studies include heterogenous groups and poorly define causes of PN (10, 25), inclusion of a homogenous group, is considered a major strength of the current study. It is important to note that though all subjects of this study received the same diagnosis, variability in iatrogenic and/or traumatic events, as well as the nerves affected by the events differed between patients. This is also the reason that the lead was placed slightly different in each patient. Further, the current analysis does not correct for confounders such as pain medication intake, follow-up, age, gender, type of electrode, waveforms and pain score at baseline, hence why conclusions should be taken with caution. Moreover, SCS is also shown to have an effect on quality of life (67), however, as this is not evaluated in the current study, this would form an interesting field for future research. Further, as this study is retrospective, there are no matching controls, hence why the placebo effect could not be properly evaluated. The current analysis does not measure duration between nerve injury and implantation of SCS, which may be a relevant topic for prospective studies as it is suggested that early SCS, within 24 h after nerve injury, is related to higher rate of responders and longer duration of analgesic effects compared to late SCS, 16 days after nerve injury (68). Future research including homogenous groups of PN, variable follow-up moments, standardized surgical approach and sham-controls are necessary, as these may lead to a better understanding of pain pathophysiology and SCS mechanisms.

## 5 Conclusion

Traumatic and iatrogenic PN places a huge burden on patients' lives, especially in case they are refractory to conventional pain strategies. Current outcomes highlight SCS as a promising treatment option for intractable traumatic and/or iatrogenic PN. Future research involving homogeneous cohorts of peripheral neuropathy patients and the use of sham controls will be crucial to further explore the clinical potential of SCS for traumatic and iatrogenic neuropathic pain. In parallel, prospective trials directly comparing SCS with treatments such as PNS are essential to validate its efficacy, support the expansion of its indications, and guide its integration into clinical guidelines for managing intractable peripheral neuropathy.

## Data Availability

The original contributions presented in the study are included in the article/Supplementary Material, further inquiries can be directed to the corresponding author.
